# Accuracy and Quality Assessment of EUS-FNA: A Single-Center Large Cohort of Biopsies

**DOI:** 10.1155/2012/139563

**Published:** 2012-10-31

**Authors:** Benjamin Ephraim Bluen, Jesse Lachter, Iyad Khamaysi, Yassin Kamal, Leonid Malkin, Ruth Keren, Ron Epelbaum, Yoram Kluger

**Affiliations:** ^1^Technion-Israel Institute of Technology, The Bruce and Ruth Rappaport Faculty of Medicine, Israel; ^2^Technion-Israel Institute of Technology and EUS Service, The Bruce and Ruth Rappaport Faculty of Medicine, Rambam Healthcare Campus, Haifa, Israel; ^3^Departments of Gastroenterology and Community Medicine, Rambam Health Care Campus, Haifa, Israel; ^4^Oncology Ambulatory Care, Rambam Healthcare Campus, Haifa, Israel

## Abstract

*Introduction*. Thorough quality control (QC) study with systemic monitoring and evaluation is crucial to optimizing the effectiveness of EUS-FNA. *Methods*. Retrospective analysis was composed of investigating consecutive patient files that underwent EUS-FNA. QC specifically focused on diagnostic accuracy, impacts on preexisting diagnoses, and case management. *Results*. 268 patient files were evaluated. EUS-FNA cytology helped establish accurate diagnoses in 92.54% (248/268) of patients. Sensitivity, specificity, PPV, NPV, and accuracy were 83%, 100%, 100%, 91.6%, and 94%, respectively. The most common biopsy site was the pancreas (68%). The most accurate location for EUS-FNA was the esophagus, 13/13 (100%), followed by the pancreas (89.6%). EUS-FNA was least informative for abdominal lymph nodes (70.5%). After FNA and followup, eight false negatives for tumors were found (3%), while 7.5% of samples still lacked a definitive diagnosis. *Discussion*. QC suggests that the diagnostic accuracy of EUS-FNA might be improved further by (1) taking more FNA passes from suspected lesions, (2) optimizing needle selection (3) having an experienced echo-endoscopist available during the learning curve, and (4) having a cytologist present during the procedure. QC also identified remediable reporting errors. In conclusion, QC study is valuable in identifying weaknesses and thereby augmenting the effectiveness of EUS-FNA.

## 1. Introduction

Endoscopic ultrasound has proven to be a highly sensitive tool for diagnosing lesions in and adjacent to the gastrointestinal tract [1]. Aspirated samples can be sent for histological, cytological, and chemistry analyses, all of which may establish a lesion/tumor as being benign or malignant, without the need for more invasive interventions. Recent surveys have indicated that EUS-FNA has been well received by the medical community at large [2]. While it has been seen that EUS-FNA can effectively establish diagnoses, it is still a relatively new procedure with overall effects on patient management that remain to be fully investigated.

FNA, originally used by Martin and Ellis [3] to diagnose suspected neoplasms, was performed under the guidance of palpation. Newer technology has allowed real-time FNA to be performed under EUS guidance using various 19–25 gauge needles [4], which has markedly increased its accuracy [5] in detecting and staging lesions while evaluating the surrounding lymph and vascular networks [6]. Bentz et al. [7] summarized that real-time EUS-FNA makes possible the accurate definitive diagnosis of pancreatic, mediastinal, and retroperitoneal masses as malignancies by acquiring tissue at primary sites and via relevant lymph node and hepatic analyses. Establishing proper staging by EUS-FNA has led to a clinically significant decrease in immediate futile surgical operations being performed, opting instead for a more appropriate palliative or neoadjuvant chemoradiation therapy for advanced cancers [8]. Also, EUS staging of tumors at the T1 level leads to endoscopic curative resections, again obviating unnecessary operations. 

EUS-FNA has proven very useful diagnostically, obviating unwarranted procedures, and reduction of cost, all of which lead to improvements in overall patient care [9]. In studies byAlhayaf et al. [10] and Lachter et al. [11], EUS has been shown to be a valuable tool for diagnosing choledocholithiasis, demonstrating greater than 90% sensitivity and negative predictive values for stones in the biliary ducts. This obviates the alternative procedure, endoscopic retrograde cholangiopancreatography (ERCP), except for therapeutic usage as it is associated with far more complications than EUS. In another case, a patient presented with what was originally diagnosed as pancreatic cancer by CA 19–9 over 1000 ng/mL and negative hepatobiliary CT scan. However, EUS revealed a gallbladder adenocarcinoma [12], leading to successful surgical removal of the tumor. 

The focus of this research was to continue the progress of the aforementioned studies of EUS-FNA affecting patient management in a study not limited to specific GI tract lesions. This large single-center quality control study investigated how EUS-FNA impacted patient care at one hospital and how its implementation might be improved to further increase its diagnostic accuracy. Such results might help to persuade the medical community at large to utilize EUS-FNA to efficiently obtain accurate diagnoses that can lead to speedier patient recovery, fewer unnecessary operations, reduced patient and hospital medical expenses, and, most important of all, lead to better patient care. 

## 2. Materials and Methods

A retrospective clinical analysis was performed, followed by statistical analyses. 

### 2.1. Research Population

Subjects for this research consisted of two hundred sixty-eight consecutive patients from computerized case files from 2008–2010 at Rambam Healthcare Campus in Haifa, Israel. These subjects were chosen from the hospital's gastrointestinal and cytology departments, all of whom had undergone EUS-FNA by Rambam gastroenterologists and have had cytological analysis performed. This population is representative of the population of Haifa, its immediate surroundings, and various communities of northern Israel. 

### 2.2. Variables

Patient files were analyzed for management and diagnosis before and after EUS-FNA. Any change in diagnosis and/or treatment was noted, such as more aggressive or more conservative, more or fewer tests being performed, with chemotherapy and surgery among the various possibilities. Moreover, methods to improve the diagnostic accuracy of EUS-FNA were considered as to limit potential errors such as inadequate FNA samples or morbidities associated with EUS-FNA. Demographics including age and gender were noted.

### 2.3. Research Methods

Data was collected from patient files and results were charted according to the target region of EUS-FNA aspiration and also according to overall results. Data was arranged into pre-EUS-FNA and postaspiration groups as described in Hirdes et al. [13] and Anand et al. [4]. Post-EUS-FNA results were analyzed to see if the FNA had any positive or negative impact on patient care in regard to its sensitivity, specificity, and its ability to withdraw sufficient material from lesions to be effective diagnostically. Statistical analysis was accomplished in collaboration with the hospital ward quality control (QC) department. Descriptive statistics including mean and standard deviation were performed for multiple variables in the study such as demographics. Sensitivity and specificity values were determined based upon the lesion status prior to and after EUS-FNA analyses by the use of SPSS version 18 program. *P* values less than 0.05 were indicated as statistically significant. 

## 3. Results

**Figure 1:**
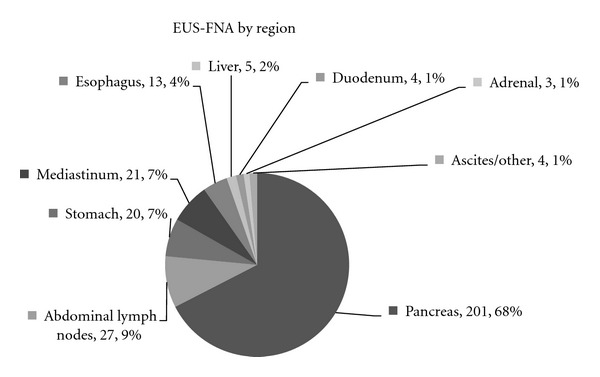
The chart represents the frequency EUS-FNA biopsy at analyzed regions. The pancreas was the most frequently biopsied organ.

A total of 268 patients' files comprised the study sample. The mean patient age was 66.6 years old. The majority (68%) of FNA samples were taken from the pancreas, with other frequent targets being the stomach, mediastinum, and abdominal lymph nodes (Figure 1). EUS-FNA diagnostic accuracy was found to be highest (100%) in the stomach and esophagus, while achieving 92.0%, 90.5%, and 74.1% accuracies in diagnosing pancreatic, mediastinal, and abdominal lymph lesions, respectively (Table 1). 

**Figure 2:**
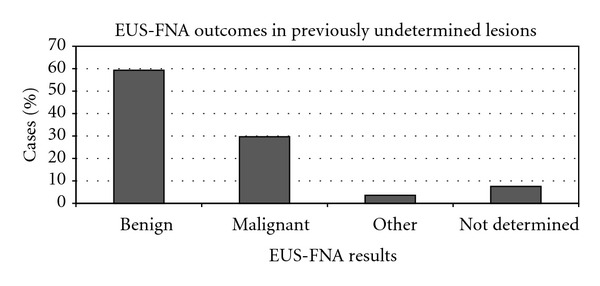
Charted is a breakdown of the diagnoses after EUS-FNA, cases of which prior diagnoses initially were undetermined. This population represented the majority of patients (84.3%, 226/268).

226 of the total 268 patients (84.3%) lacked a definitive diagnosis prior to performing EUS-FNA. Examples commonly encountered were obstructive jaundice or a widened Wirsung duct that was found on computerized tomography (CT). After EUS-FNA, 134 of the cases were determined to be benign (59.3%), 67 cases of malignancy were found (29.6%), and 7.5% of cases still lacked a definitive diagnosis following the procedure (Figure 2). 

EUS-FNA cytology proved useful in establishing the diagnosis in 248 out of 268 patients (92.54%). Sensitivity and specificity were established by evaluating diagnoses for changes prior to and after EUS-FNA. Prior to FNA, 27 of the 268 patients (10.1%) were determined to have benign lesions, 12 (4.5%) patients had malignant conditions, and 3 patients (1.1%) had lesions of chronic inflammation designed as “other.” Positive values were indicated as malignant, and negative values were labeled as benign. 

EUS-FNA diagnosed 10 out of 10 malignant cases for 100% positive predictive value (PPV). The PPV value indicates that 100% of cases having known malignancy tested positive for malignancy after EUS-FNA, indicating the importance of a positive result that EUS-FNA is diagnostic. This also indicates 100% specificity in ensuring no false positives, meaning that a lesion found to be malignant by EUS-FNA had a 100% of being malignant. EUS-FNA did not detect two other cases of malignancy, instead giving false negatives results that resulted in 83.3% (10/12) sensitivity. False negatives represent lesions that EUS-FNA cytology determined as benign, but were soon after diagnosed as malignant. Therefore, the 83.3% sensitivity indicates that a negative result by EUS-FNA has an 83.3% chance of being benign, but does not always rule out a malignant condition (see Table 3). Similarly, the NPV is an indicator for negative results. Out of 27 presumed to be benign cases prior to FNA, 22 cases were indeed benign by FNA, whereas 2 malignant cases were found: one case of chronic inflammation and two not determined. As the NPV of EUS-FNA is 91.6% (22/24), there is over a 90% chance that a negative result from EUS-FNA will indeed be a benign lesion. The overall accuracy of EUS-FNA was found to be 94.1%, which implies that 94% of the results are correct diagnostically, no matter if the result is benign or malignant (Table 3). This compares to a recent 2011 review of previous studies of diagnosing pancreatic solid masses by EUS-FNA since 1992 that established a sensitivity, specificity, PPV, NPV, and accuracy of 78–95%, 75–100%, 98–100%, 46–80%, and 78–95% [14].

The greatest percentage of false negatives or nondiagnostic FNAs was found in analysis of abdominal lymph nodes. 27 cases were identified in which EUS-FNA from abdominal lymph nodes was performed, two-thirds of which involved additional sites of EUS-FNA. Of these 27 patients, seven cases of nondiagnostic or false negative were found (25.9%), The majority of such nondiagnostic or false negative cases (6/7) of abdominal lymph nodes either involved FNA the nodes alone or with accompanying FNA from the pancreas (Table 2). 

A total of 20 cases (7.5% of total) nondiagnostic and false negative FNA cases were identified. Eight of the twenty cases were false negatives; zero false positives were found. The column labeled “FNA nondiagnostic or false negative” includes the cases in which EUS-FNA and/or subsequent cytology could not effectively diagnose a suspected lesion or resulted in a false negative (Table 1). A false negative indicates that cytological analysis of EUS-FNA aspirate showed a benign result, yet further histological analysis during surgical removal of the lesion found a malignancy. Causes included the number of FNA passes, the character of the lesion, the type and gauge number of the needle, and the experience of the performing endoscopist (one senior operator performed or attended greater than 90% of the EUS-FNA procedures). See Discussion in regard to the row titled “cysts with CEA >192 ng/mL” (Table 3). During data analysis, it was found that patient files often did not completely detail the stage of lesions prior to FNA and also did not provide data about chemotherapeutic treatment when diagnosed with a malignant condition by EUS-FNA. 

## 4. Discussion

EUS-guided FNA can have a profound influence on patient management. Its diagnostic ability is one of its greatest assets. Patients with benign conditions do not require intensive treatment and usually routine patient followup is needed, depending on the nature of the pathology. In contrast, the patients diagnosed with malignancies are referred for surgical resection when possible, chemo/radiation therapy, and/or appropriate palliative care. For both benign and malignant diseases, quality assessments of care may point to ways to improve the service provided to our patients. In providing accurate diagnoses, EUS-FNA helps to establish proper patient care while avoiding futile, costly, and potentially risky procedures and operations. 

In regard to location, EUS-FNA was most accurate in the esophagus, stomach, and adrenal and peri-GI tract areas (marked as “other” in the results section), demonstrating 100% accuracy. In the pancreas, which was the region of most frequent EUS-guided FNA usage (see Figure 1), 92.04% accuracy was achieved in correctly diagnosing pancreatic lesions. The present series reflects the local practice which involves EUS only through the upper gastrointestinal tract. Transrectal ultrasound and biopsies are performed by the proctologic surgeons using separate equipment and thus were not included in the present study. 

Despite being a highly sensitive and selective method, EUS-FNA cytology was not equally accurate for several analyzed regions. One such area was in the assessment of abdominal lymph nodes, where the accuracy was 74.07%. Accuracy in assessing mediastinal lesions was 90.48% (see Table 2). This is in comparison to prior studies by Nakahara et al. that used 22 gauge needles to obtain adequate specimens from undiagnosed abdominal lymphadenopathies in 93% of cases (*n* = 53) that led to a sensitivity of 94%, 100% specificity, 100% positive predictive value (PPV), 90% negative predictive value (NPV), and an overall 96% accuracy [15]. For mediastinal lesions, comparative studies (*n* = 37) demonstrated 79.3% sensitivity, 100% specificity, and 85% accuracy overall in detecting malignant mediastinal lymphandenopathies [16]. 

 Several factors can be responsible for the diminished accuracy of these selected areas. First, the number of passes made into the lesion of interest comes into question. In two out of the 20 cases (10.0%) of FNA being nondiagnostic or false negative, it was documented that three passes were made from the lesion by FNA. Five cases (25.0%) involved two passes and three cases (15.0%) were documented where only one pass was attempted to aspirate contents from suspicious lesions. This can be due to several reasons. Minimal fluid was reported to be withdrawn from eight of the 20 cases (40.0%), which likely made it difficult for cytologists to delineate or exclude a specific pathology if present (see Table 3). Moreover, minimal fluid withdrawal was a key reason found for the false negative cases seen (see bottom of Table 3). The local tendency to attempt FNA on cysts smaller than 2 cm when easily accessible for potential biopsy may lead to a high rate of inadequate fluid for evaluation. Fluid is tested by performing a 1 : 2 or 1 : 3 dilution of the aspirated material so as to maximize the ability to allow analyses of the fluid in chemistry (CEA and amylase) and cytological tests. 

Lesions with very viscous mucous contents often are unsuitable for obtaining adequate fluid for chemistry (CEA and amylase) evaluation. Data from samples taken from EUS-FNA biopsies of pancreatic cysts have shown that a CEA level above 192 ng/mL is indicative of mucinous neoplasms in 79% of cases [17]. In three of the original nondiagnostic 23 cases (13.0%), the measured CEA levels were 210, 1905, and 6740 ng/mL, respectively, which supported the diagnosis of a mucinous neoplasm or IPMN. Originally 245 out of 268 cases were diagnostic by EUS-FNA cytology (see Table 1); factoring in this diagnostic criteria adds three more cases to arrive at 248 out of 268 (92.54%) total diagnostic cases. 

Issues that infrequently complicate EUS-FNA procedures are the vicinity of the lesion for biopsy and the condition of the patient. It was documented in three nondiagnostic FNA cases (15.0%) that fewer passes were taken of suspected lesions because the operator found it difficult to insert the needle into the lesion for biopsy. In one of these cases, multiple vessels surrounded the suspected mass, resulting in a narrow window to insert the needle for FNA. This may be remedied by using newer small 25 gauge or more flexible needles that can more easily penetrate hard lesions ensuring the ease of puncture and a greater quantity of aspirate extracted [18]. In two other cases, despite the standard eight hours fast patient preprocedure preparation, the patient's stomachs were still partly full of food contents. In such cases, EUS-FNA may best be postponed and the patients instructed to fast for a longer time. However, this may also be indicative of a gastric outlet obstruction that may be in part due to tumors. Another case involved a duodenal obstruction that limited the passage of the endoscope, making it difficult for the endoscopist to pinpoint the lesion site for FNA biopsy. In addition, one EUS-FNA procedure caused a patient to bleed after one pass into the lesion, so no further attempts of performing FNA were made. Lastly, two of the cases (10.0%) that proved nondiagnostic by EUS-FNA were suspected by the operating endoscopist to be of autoimmune origin or of chronic inflammation. None of these cases manifested in being diagnostic cytologically, resulting in the need for further blood tests and biopsies. 

Occasionally, the documentation on patient case files was insufficient to determine a specific cause of nondiagnostic FNAs. There are two junior echoendoscopists currently working in Rambam Healthcare Campus, to whom may be attributed several of the nondiagnostic FNAs. One senior operator performed or attended greater than 90% of the EUS-FNA procedures. However, it cannot be confirmed if the nondiagnostic FNAs were due to operator inexperience because the patient files investigated did not list the name of the doctor who performed the respective EUS-FNA. Reports may be written by either the attending or the in-training echoendoscopist. Furthermore, recent literature showed as good results among newly trained echoendoscopists as compared to experienced self-trained ones. The number and type of needles used were rarely documented, factors which may impact the amount of material for cytology and chemical analyses. Moreover, local compensation for EUS is nationally standardized and strongly discourages usage of more than one needle. One additional method to ensure diagnostic accuracy of EUS-FNA samples is to have a cytologist present during the procedure to ensure the adequacy of the aspirated samples and has been shown in several centers to increase the accuracy of cytological diagnosis by 10–15% [19]. This has also been associated with shortening procedure time, thus theoretically reducing the risk of complications. Although this would impose somewhat increased cost and logistical burdens, a cytologist present during EUS-FNA along with further and more detailed documentation of FNAs could provide valuable insight into how important any of these aspects is when evaluating possible causes of fruitless FNA procedures. 

In analyzing the 268 patient files from 2008–2010, there was one reported case of FNA-related complication in which a minor hemorrhage occurred near the site of aspiration. This required the patient to be hospitalized for several additional hours before being released. No other cases of EUS-FNA morbidity or mortality caused by EUS-FNA were found; therefore, the morbidity rate is 1/268, or 0.37% with a mortality of 0.0%. This is in comparison to published benchmark data, which showed that the morbidity rate from EUS-guided FNA was 0.98% overall, including postprocedural pancreatitis, pain, and mortality [20]. This retrospective analysis indicates that EUS-guided FNA is a useful diagnostic procedure that minimizes potential morbidity that patients may experience. 

As previously noted, accurate diagnoses were made possible by EUS-FNA in 92.54% of evaluated cases, which minimized the overall health and financial costs brought about by the amount of extra tests, chemotherapy, and fruitless operations that patients may undergo as a result of misdiagnosis. A previous report found that EUS-FNA markedly decreased the frequency of futile surgery for pancreatic cancers while additionally aiding in the tailoring of optimal individualized patient treatment according to the stage of the patient's cancer [21]. This may also be due to the ability of EUS to visualize the surrounding lymph nodes around a lesion to determine the likely effectiveness of surgery. This overall correlates to a significant savings of time and money for patients, doctors, and hospitals alike. 

## 5. Limitations

 Limitations of this study included its retrospective nature at a single medical center. Although many of the computerized data files investigated contained fairly complete data, analysis was limited in a few files that consisted of incompletely documented reports. The study was also performed at a single healthcare center with its equipment and personnel. Therefore, it should be noted that the diagnostic accuracy of EUS-FNA may vary by several factors, including the experience of the center's echoendoscopists, cytologists, and lab personnel, and also by the sensitivity of its equipment. Rambam Healthcare Campus is also a training facility for many young doctors, and as such, there exist wide gaps in experience of the performing echoendoscopists, although a senior endoscopist supervises or consults on most procedures.

## 6. Conclusions

EUS-FNA was found to have 94.1% accuracy. Quality control analysis and literature suggest that this may be further improved by more thorough reporting of procedural information (perhaps with forcedfields in the electronic medical report), optimizing needle selection, and by having a senior echo-endoscopist and a cytologist present during each procedure. 

## Figures and Tables

**Table 1:** EUS-FNA by region and diagnostic accuracy.

Region	Frequency	FNA nondiagnostic or false negative	FNA diagnostic (%)
Pancreas	201	16	92.04
Abdominal lymph nodes	27	7	74.07
Stomach	20	0	100.00
Mediastinum	21	2	90.48
Esophagus	13	0	100.00
Liver	5	0	100.00
Duodenum	4	0	100.00
Adrenal	3	0	100.00
Ascites/other	4	1	75.00

Total	268	20	92.54

Various regions sampled by EUS-FNA cytology, showing frequency of FNA, number of false negatives or nondiagnostic samples, and overall diagnostic accuracy are displayed.

Total includes several cases of overlap in patients where multiple regions were sampled by FNA. For example, a patient had FNA biopsies taken from his pancreas and abdominal lymph nodes.

**Table 2:** FNA results for abdominal lymph nodes.

Region	Cases	FNA nondiagnostic or false negative	Percent of FNA diagnostically useful
Nodes alone	9	3	66.67
With pancreas	8	3	62.50
With stomach	2	0	100.00
With liver	2	0	100.00
With adrenal	1	0	100.00
With mediastinum	3	0	100.00
With mediastinum and pancreas	1	1	0.00
With other (ascites)	1	0	100.00

Total	27	7	70.47

Displayed in this chart are the various regions from which EUS-guided FNA was performed on abdominal lymph nodes either alone or in combination with other organs. Alongside each value is the number of cases that the FNA proved unhelpful (nondiagnostic or false negative) in establishing the patient's diagnosis.

**Table 3:** nondiagnostic EUS-FNA cases.

Reason	Frequency	Percent of total
Number of FNA passes		
3 passes	2	10.00
2 passes	5	25.00
1 pass	3	15.00
Not listed	10	50.00

Total	20	100.00

Other reasons		
Unidentifiable cause	10	50.00
Minimal fluid withdrawn	8	40.00
Difficult to pass needle*	3	15.00
Suspected autoimmune or chronic inflammation	2	10.00
False negatives	8	40.00

Total	20	100.00

Sensitivity	83.3%	
Specificity	100.00%	
Positive predictive value	100.00%	
Negative predictive value	91.6%	
Accuracy	94.1%	

Listed is a summary of suspected causes of nondiagnostic and false negative FNA cases, along with sensitivity, specificity, PPV, NPV, and overall accuracy of EUS-FNA.

*The category labeled “difficult to pass needle” is a subgroup of the cases that fall under the group “minimal fluid withdrawn.”
